# An increasing tendency of precocious puberty among Korean children from the perspective of COVID-19 pandemic effect

**DOI:** 10.3389/fped.2022.968511

**Published:** 2022-08-25

**Authors:** Kyu Hee Choi, Seung Chan Park

**Affiliations:** Highki Growth Clinic, Seoul, South Korea

**Keywords:** precocious puberty (PP), early puberty, COVID-19, pandemic, social distancing

## Abstract

**Introduction:**

This study was conducted to investigate changes and new trends over the past 6 years by analyzing the current status of precocious puberty (PP) treatment and treatment costs in Korea between 2016 and 2021.

**Materials and methods:**

Annual and monthly number of patients diagnosed with PP from 2016 to 2021 were reviewed using the data from Healthcare Bigdata Hub. Annual medical insurance expenses for the treatment of PP were also reviewed. The data were compared by the gender of the patients.

**Results:**

The number of patients diagnosed with PP rose from 86,352 in 2016 to 166,645 in 2021, while medical expenses rose from KRW 64,111,689,000 in 2016 to KRW 134,642,100,000 in 2021. The percentage of male PP patients increased from 9.21% in 2016 to 19.55% in 2021.

**Conclusion:**

Increasing numbers of Korean patients diagnosed with PP. Consistent with the situation in other countries, the rapid increase in the number of cases since April 2020 appears to be a result of the COVID-19 pandemic. In Korea, this is considered a nationwide phenomenon. Also on the rise is the incidence of PP in males, which appears to be due to an increased awareness of the phenomenon. Further investigations are required to determine the possible causes in increasing prevalence of PP.

## Introduction

When secondary sex characteristics appear before the age of 8 in females and 9 in males, this is referred to as precocious puberty (1). Since the 1970s, the onset of secondary sex characteristics has sped up by 0.24 years every decade on a global scale (2). This early onset of puberty is a sign of socioeconomic and environmental change. Although the number of children and adolescents in Korea is declining, the number of patients diagnosed with precocious puberty and the associated costs continue to rise (3). Kim et al. demonstrated that the incidence of precocious puberty rose from 2004 to 2010 (4), and Kim et al. demonstrated that the prevalence of precocious puberty rose steadily and dramatically from 2008 to 2014 (5). Since then, 8 years have passed, and the world is undergoing rapid social change as a result of the COVID-19 pandemic. Among these changes, since the COVID-19 pandemic in Italy, Turkey, India, China, etc., the number of patients with precocious puberty increased significantly, according to the results released (6–12). This study was conducted to observe the trends of annual and monthly number of patients with precocious puberty and their medical expenses in Korea from 2016 to 2021.

## Materials and methods

Based on the census (13) of the national statistical portal (Korean Statistical Information Service, KSIS), the number of youth (0–19 years old) by year and the ratio of the youth population to the total population were investigated. The data for 2021 was derived from population projections for the future (14).

Every patient who visits hospital in Korea are registered with diagnoses according to the Korean Classification of Disease 10th revision (KCD-10). We investigated the data of the Healthcare Big Data Hub (15) registered as E301 (Early puberty, according to KCD-10). The number of patients diagnosed with precocious puberty by year, month, gender, and age in increments of 5 years, and region, as well as the status of insurance treatment costs, were analyzed.

## Results

### Domestic changes in youth population by year

The youth population in Korea decreased from 9,906,000 in 2016 to 8,490,000 in 2021. The ratio of the youth population to the total population also decreased from 19.32% in 2016 to 16.41% in 2021 (Table 1).

**TABLE 1:** Korea adolescent population aged 0–19 (in thousands).

	2016	2017	2018	2019	2020	2021*
Total population	51,270	51,423	51,630	51,779	51,829	51,744
Population under 19	9,906	9,605	9,316	9,030	8,704	8,490
Percentage of population under 19 (%)	19.32	18.68	18.04	17.44	16.79	16.41

*Projected population figures.

### Status of patients with precocious puberty by year

**FIGURE 1:**
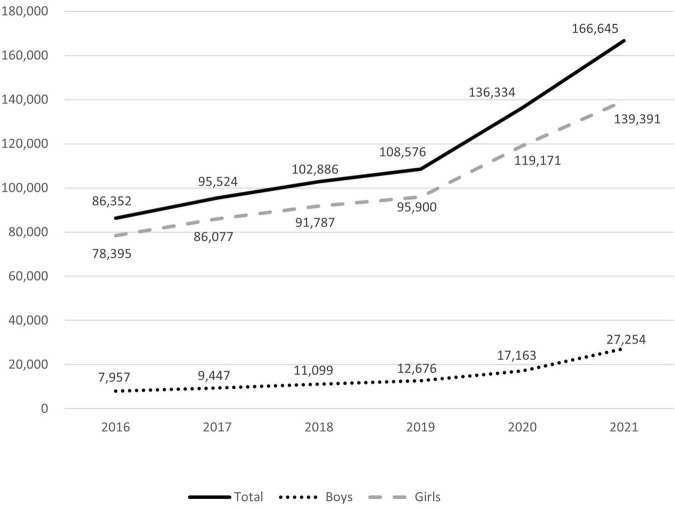
Annual number of patients diagnosed with precocious puberty.

**FIGURE 2:**
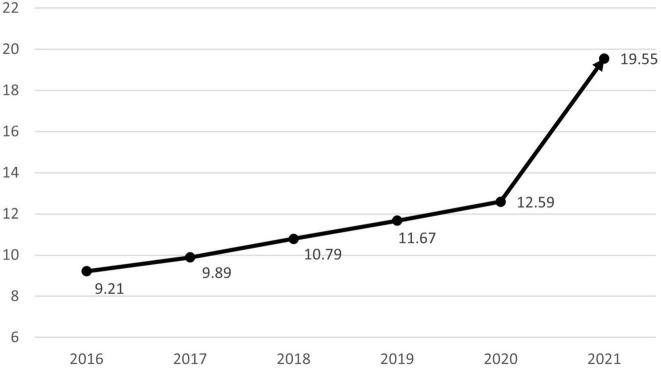
Increasing tendency for boys diagnosed with precocious puberty compared to girls (%).

**FIGURE 3:**
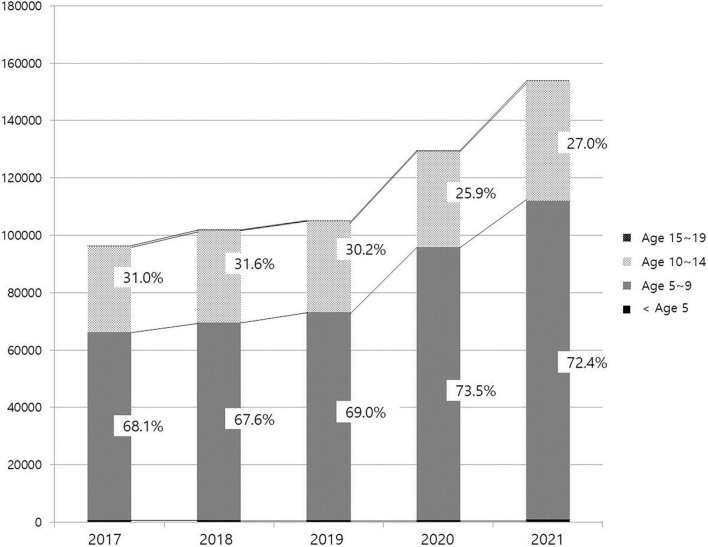
Age distribution of female patients with precocious puberty.

**FIGURE 4:**
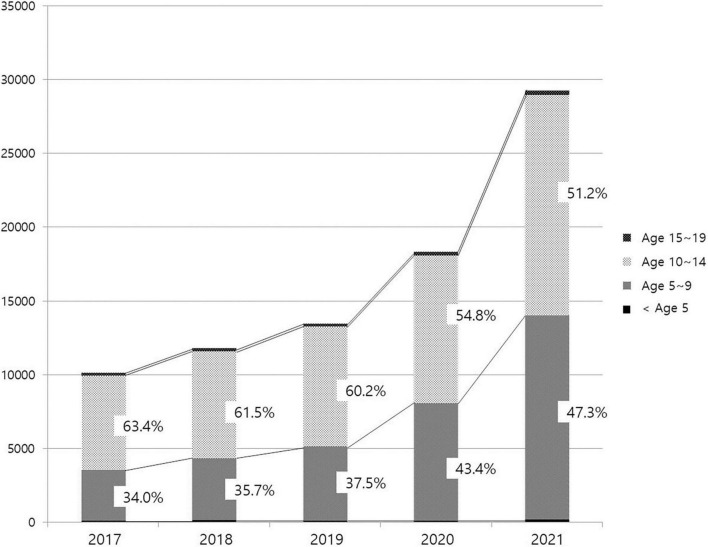
Age distribution of male patients with precocious puberty.

The number of patients treated in medical facilities for E301 increased 1.93-fold between 2016 and 2021, from 86,352 to 166,521 (Figure 1). From 7,957 to 27,254, the number of male patients increased 3.43-fold, and the number of female patients increased 1.78-fold from 78,395 to 139,391. The proportion of male patients increased from 9.21% in 2016 to 19.55% in 2021 (Figure 2). The highest portion of female patients was in the 5-9-year age group, while the highest proportion of male patients was in the 10-14-year age group (Figures 3, 4).

### Status of patients with precocious puberty by region

**FIGURE 5:**
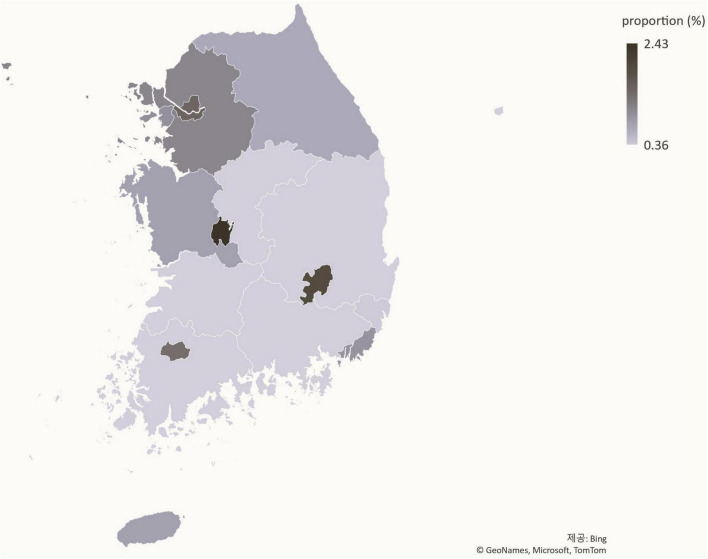
Population distribution of precocious puberty by region in 2016 (%).

**FIGURE 6:**
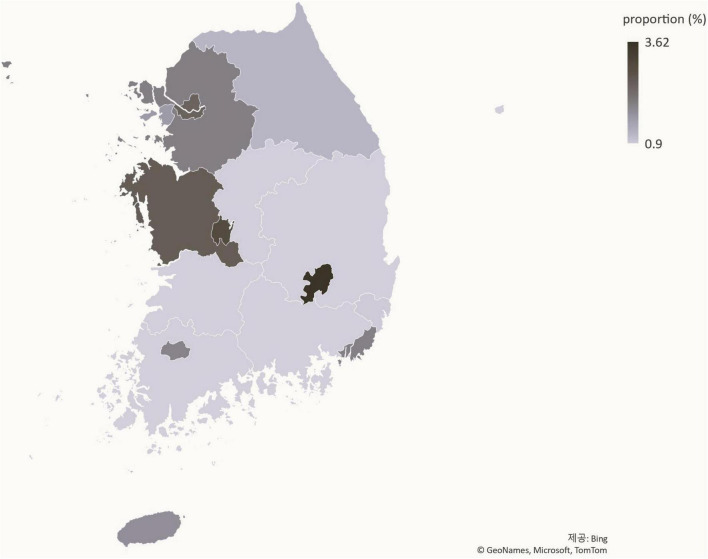
Population distribution of precocious puberty by region in 2021 (%).

The ratio of patients with precocious puberty to the total number of youths has increased nationally between 2016 (Figure 5) and 2021 (Figure 6). Daegu had the highest proportion of patients with precocious puberty.

### Monthly status of patients with precocious puberty

**FIGURE 7:**
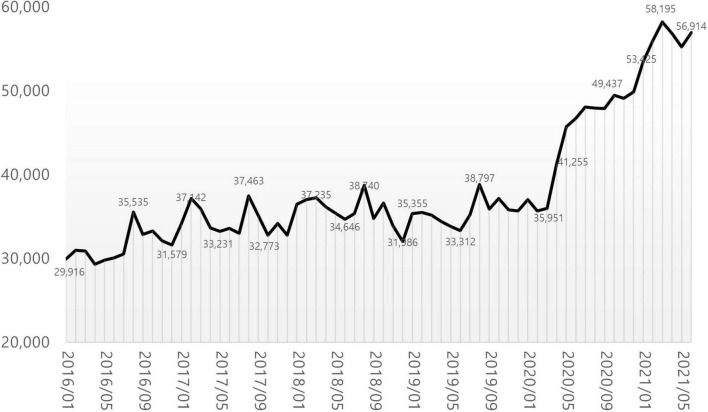
Monthly number of patients diagnosed with precocious puberty.

29,916 patients were treated at medical institutions for E301 during the month of January 2016. The number stayed in the 30,000’s for 4 years and 2 months until March 2020, a 1.2-fold increase. In April 2020, the number increased 1.5-fold compared to the previous month, reaching 41,255. A year later, in March 2021, the number reached 58,195, a 1.68-fold increase compared to January 2016 (Figure 7).

### Cost of treatment for precocious puberty

The total cost of medical care for E301 patients treated at medical institutions increased 1.99-fold from KRW 43,579,409,000 in 2016 to KRW 86,846,620,000 in 2021. Likewise, the costs of accession deductible payment increased 2.33-fold, from KRW 20,532,280,000 to KRW 47,795,480,000 (Table 2).

**TABLE 2:** Annual medical insurance expenses for precocious puberty (KRW 1000 won).

	2016	2017	2018	2019	2020	2021
Medical care benefits	43,579,409	49,264,944	52,220,670	50,846,925	65,078,828	86,846,620
Accession deductible payment	20,532,280	23,650,423	25,948,170	26,181,533	35,033,886	47,795,480
Total expenses	64,111,689	72,915,367	78,168,840	77,028,458	100,112,714	134,642,100

## Discussion

Secondary sex characteristics, rapid skeletal maturation, and rapid growth appear during puberty (16). Hormones of the hypothalamus-pituitary-gonadal axis (HPG axis) exist at the center of this change. The pituitary gland secretes follicle stimulating hormone (FSH) and luteinizing hormone (LH) in response to hypothalamic gonadotropin-releasing hormone (GnRH) stimulation. This leads to gonadal secretion of testosterone in males, and estradiol (E2) in female, resulting in the fertility of both sexes (17). Since hormones are sensitive to changes in both the external environment and the genetic unit, puberty has been a significant socioeconomic milestone.

**FIGURE 8:**
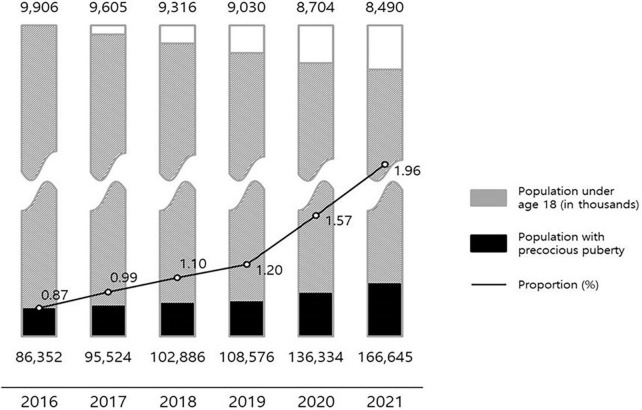
Annual number of patients diagnosed with precocious puberty compared with population.

Using statistical data from the national statistical portal and the Healthcare Big Data Hub, this study analyzed the medical status of patients with precocious puberty in Korea from 2016 to June 2021. Compared to 2016, the number of patients diagnosed with precocious puberty increased 1.93-fold in 2021. During the same time period, the population of youth aged 0–19 decreased, and the proportion of adolescents diagnosed with precocious puberty increased 2.25-fold, from 0.87% in 2016 to 1.96% in 2021, a greater increase than the increase in the number of patients (Figure 8). In addition, the ratio of patients with precocious puberty to the population is rising in nearly all regions, indicating that the rise in the number of patients with precocious puberty is a nationwide phenomenon. As the number of patients increases, so do the costs associated with precocious puberty. The total cost of medical care and the amount of accession deductible payment increased 2.1-fold between 2016 and 2021, from KRW 64,111,689,000 to KRW 134,642,100,000. Considering previous research (3), it is believed that the number of patients with precocious puberty in Korea continues to rise.

In this study, the authors discovered two new trends. One is the significant increase in patients seen after April 2020. The growth rate between 2019 and 2021 is steeper than the change between 2016 and 2019, and a monthly analysis reveals a meteoric rise beginning in April 2020. This trend continued until June 2021. After the first COVID-19 outbreak in China at the end of 2019, the world entered a crisis, and each country implemented lockdowns or equivalent measures in 2020, making telecommuting and remote learning the norm, and outdoor activities restricted. Changes in lifestyle have caused an increase in endocrine diseases (18). In Italy, outpatient treatment for suspected symptoms of early puberty and precocious puberty increased dramatically compared to the same time period in 2019 (7). In addition, the phenomenon of accelerated puberty onset was identified, and the rate of precocious puberty diagnosis increased (6). Compared to the 3 years preceding the pandemic, the diagnosis rate of idiopathic central precocious puberty (ICPP) in Turkey has more than doubled (8). Both central precocious puberty (CPP) and rapidly progressive early puberty (RPEP) increased by greater than threefold in other studies (10). After lockdown, the referrals for precocity and the number of children diagnosed with ICPP quadrupled in India (11). The prevalence of precocious puberty in Shanghai, China also has more than doubled (12). It is suggested that excessive use of digital devices is the primary cause of this phenomenon. In a study conducted between October 2020 and March 2021, the salivary melatonin levels of 39 females diagnosed with precocious puberty were significantly lower than those of the control group. This was attributed to the effects of light stimulation and electromagnetic fields (EMF) generated by electronic devices (19). Low melatonin levels affect the HPG axis, thereby accelerating the onset of puberty (20). In addition, restriction of physical activity and increased BMI due to high-calorie food consumption, fear of the pandemic, mental factors such as anxiety, changes in sleep patterns, exposure to environmental hormones, and direct stimulation of the central nervous system due to COVID-19 infection are cited as potential causes (21, 22). The situation in Korea is similar. After the first COVID-19 patient was reported (23) in January 2020, strict social isolation measures were implemented, school was suspended until May 2020, and some classes are still conducted remotely. According to a study (24) conducted by the Department of Pediatrics at Eulji University Hospital, the proportion of overweight and obese children who presented with precocious puberty in 2020 was significantly higher than in 2019. This was more prevalent in males. Moreover, while the overall number of pediatric outpatients has been decreasing, the number of outpatients due to short stature, precocious puberty, and obesity is increasing, and this is thought to be due to the decrease in physical activity as a result of the COVID-19 pandemic. In this study we confirmed that the number of patients diagnosed with precocious puberty increased from April 2020 to April 2021 in Korea. We could speculate several possible factors for this trend based on the previous studies. First, increasing obesity due to the lack of physical activities could be the main factor. It has been suggested that obesity, fast food consumption, and the consumption of growth-related health functional foods are factors accelerating the onset of puberty in Korean adolescents (25). The BMI of elementary school students has risen significantly over the past year, as the physical activity of adolescents has decreased, and their consumption of fast foods has increased as a result of COVID-19 (26). Meanwhile, the immune-related health functional food industry is experiencing a boom that is unprecedented (27). Under these conditions, it appears inevitable that the number of patients receiving treatment for precocious puberty will increase rapidly. Second, overuse of digital devices during the social distancing and remote learning period could affects this phenomenon, too. According to the Korea Information Society Development Institute’s “Comparison of Media Usage Behaviors of Adolescents Before and After COVID-19” report (28), the use of paper media decreased as a result of online classes, while time spent on laptops, tablets, desktops, and smartphones increased. Third, endocrine-disrupting chemicals are possible factor of this tendency. Since social distancing caused non-contact delivery culture, it is also necessary to observe a correlation between the rapid increase in the use of disposable items such as plastic and vinyl and precocious puberty (29). These chemicals are known to the factors that lead to precocious puberty. All of these factors working together, precocious puberty patients are steeply increasing during the pandemic in Korea.

An increasing proportion of males are diagnosed with precocious puberty, which is another trend. Since 2016, the proportion of males, who accounted for less than 10% in the previous study, has steadily risen, reaching 12.59% in 2020 and 19.55% in 2021. Particularly, the rate of medical treatment among 5 to 9-year-olds increased from 34 to 47.3%, which appears to have contributed to the rise in male patients. Existing studies show that the precocious puberty is 10 times more prevalent in females than in males; however, severe cases accompanied by abnormalities of the central nervous system, such as malformations, tumors, or inflammation, are more prevalent in males; therefore, referral to a tertiary medical institution has been deemed necessary (13, 30). However, according to an analysis of male patients with precocious puberty in Korea from 2001 to 2016 by Lee et al. (31) in 2018, the rate of idiopathic precocious puberty, for which it is difficult to identify a specific cause, has increased with the annual increase in the number of patients, accounting for more than 60%. After the age of eight, the proportion increases even further. These results are attributed to an increase in parental awareness through the Internet and broadcast media, in addition to environmental factors such as nutritional improvement and endocrine disrupting substances. Males, unlike females, have an unclear onset of secondary sex characteristics, making it easy to miss the opportunity to diagnose precocious puberty. In this study, the increase in the proportion of male patients before 9 years of age and the total number of male patients occurred simultaneously, which means, the treatment rate has increased as a result of the growing awareness of precocious puberty in males. In addition, the sudden increase in male patients coincided with the period of COVID-19 social distancing. As mentioned previously, this is likely due to the fact that the restrictions on physical activity and tendency toward obesity caused by the pandemic are more pronounced in previously active males. This supports the possibility that the increase in the number of patients with precocious puberty during this time period is due to changes in lifestyle brought about by the COVID-19 pandemic.

The trend of precocious puberty in Korea over past 6 years was analyzed by year, gender, age, and region in this study. Through this process, it was possible to confirm the need for a large-scale correlation study on various factors that can influence precocious puberty. In addition, Korea is a country where Western medical treatment for precocious puberty utilizing GnRH agonist and oriental medical treatment (32) utilizing herbal medicine to regulate hormones coexist. The majority of non-insured patients receiving oriental medical treatment are omitted from this study’s statistics. In the future, research should be conducted on the current status of treatment for precocious puberty by various medical institutions and the effects thereof.

## Data availability statement

The original contributions presented in the study are included in the article/supplementary material, further inquiries can be directed to the corresponding author.

## Ethics statement

Ethics review and approval/written informed consent was not required as per local legislation and institutional requirements.

## Author contributions

Both authors listed have made a substantial, direct, and intellectual contribution to the work, and approved it for publication.

## References

[1] Muir A. Precocious puberty. *Pediatr Rev.* (2006) 27:373–81. 10.1542/pir.27-10-373 17012487

[2] Eckert-Lind C Busch AS Petersen JH Biro FM Butler G Brauner EV Worldwide secular trends in age at pubertal onset assessed by breast development among females: a systematic review and meta-analysis. *JAMA Pediatr.* (2020) 174:e195881. 10.1001/jamapediatrics.2019.5881 32040143 PMC7042934

[3] Choi KH Park SC. An increase of patients diagnosed as precocious puberty among Korean children from 2010 to 2015. *J Pediatr Korean Med.* (2016) 30:60–5. 10.7778/jpkm.2016.30.4.060

[4] Kim SH Huh K Won SH Lee KW Park MJ. A significant increase in the incidence of central precocious puberty among Korean females from 2004 to 2010. *PLoS One.* (2015) 10:e141844. 10.1371/journal.pone.0141844 26539988 PMC4634943

[5] Kim YJ Kwon AR Jung MK Kim KE Suh JW Chae HW Incidence and prevalence of central precocious puberty in Korea: an epidemiologic study based on a national database. *J Pediatr.* (2019) 208:221–8. 10.1016/j.jpeds.2018.12.022 30857777

[6] Stagi S Masi S Bencini E Losi S Paci S Parpagnoli M Increased incidence of precocious and accelerated puberty in females during and after the Italian lockdown for the coronavirus 2019 (COVID-19) pandemic. *Ital J Pediatr.* (2020) 46:165. 10.1186/s13052-020-00931-3 33148304 PMC7609833

[7] Verzani M Bizzarri C Chioma L Bottaro G Pedicelli S Cappa M. Impact of COVID-19 pandemic lockdown on early onset of puberty: experience of an Italian tertiary center. *Ital J Pediatr.* (2021) 47:52. 10.1186/s13052-021-01015-6 33673836 PMC7935003

[8] Acar S Ozkan B. Increased frequency of idiopathic central precocious puberty in girls during the COVID-19 pandemic: preliminary results of a tertiary center study. *J Pediatr Endocrinol Metab.* (2022) 35:249–51. 10.1515/jpem-2021-0565 34881532

[9] Akyurek N. Evaluation of COVID-19 pandemic process effect on the increase of precocious puberty and premature thelarche. *Genel Top Derg.* (2022) 32:32–5. 10.54005/geneltip.1011301

[10] Acinikli KY Erbas IM Besci O Demir K Abaci A Bober E. Has the frequency of preccocious puberty and rapidly progressive early puberty increased in girls during the COVID-19 pandemic? *J Clin Res Pediatr Endocrinol.* (2022). 10.4274/jcrpe.galenos.2022.2022-12-11 [Epub ahead of print]. 35633642 PMC9422906

[11] Shruti AM Chriantap O Vaman K Nikhil S Ketan G Neha K Impact of COVID-19 lockdown on idiopathic central precocious puberty – experience from an Indian centre. *J Pediatr Endocrinol Metab.* (2022) 35:895–900. 10.1515/jpem-2022-0157 35658967

[12] Chen Y Chen J Tang Y Zhang Q Wang Y Li Q Difference of precocious puberty between before and during the COVID-19 pandemic: a cross-sectional study among Shanghai school-aged girls. *Front. Endocrinol.* (2022) 13:839895. 10.3389/fendo.2022.839895 35392135 PMC8979840

[13] Korean Statistical Information Service. *Population Census.* (2020). Available online at: https://kosis.kr/statHtml/statHtml.do?orgId=101&tblId=DT_1IN1503&conn_path=I2 (https://kosis.kr/statHtml/statHtml.do?orgId=101&tblId=DT_1IN1503&conn_path=I2) (accessed July 29, 2021).

[14] Korean Statistical Information Service. *Population Projection for Korea.* (2021). Available online at: https://kosis.kr/statHtml/statHtml.do?orgId=101&tblId=DT_1BPA001&conn_path=I2 (https://kosis.kr/statHtml/statHtml.do?orgId=101&tblId=DT_1BPA001&conn_path=I2) (accessed December 09, 2021).

[15] Healthcare Bigdata Hub. *Statistics by Four-character Subcategories.* (2021). Available online at: http://opendata.hira.or.kr/op/opc/olap4thDsInfo.do (http://opendata.hira.or.kr/op/opc/olap4thDsInfo.do) (accessed November 01, 2021)

[16] Bradley SH Lawrence N Steele C Mohamed Z. Precocious puberty. *BMJ.* (2020) 368:I6597. 10.1136/bmj.l6597 31932347

[17] Spaziani M Tarantino C Tahani N Gianfrilli D Sbardella E Lenzi A Hypothalamo-pituiary axis and puberty. *Mol Cell Endocrinol.* (2020) 520:111094. 10.1016/j.mce.2020.111094 33271219

[18] Peinkhofer M Bossini B Penco A Giangreco M Pellegrin MC Vidonis V Reduction in pediatric growth hormone deficiency and increase in central precocious puberty diagnoses during COVID 19 pendemics. *Ital J Pediatr.* (2022) 48:49. 10.1186/s13052-022-01238-1 35346309 PMC8960104

[19] Stagi S Ferrari M Paiusco G Moriondo M Azzari C. Possible role of melatonin in precocious and accelerated puberty in females during the COVID-19 pandemic. *Ital J Pediatr.* (2021). 10.21203/rs.3.rs-855928/v1 [Epub ahead of print]. PMC7609833 33148304

[20] de Holanda FS Tufik S Bignotto M Maganhin CG Vieira LHL Baracat EC Evaluation of melatonin on the precocious puberty: a pilot study. *Gynecol Endocronol.* (2011) 27:519–23. 10.3109/09513590.2010.501888 20642379

[21] Umano GR Maddaluno I Riccio S Lanzaro F Antignani R Giuliano M Central precocious puberty during COVID-19 pandemic and sleep disturbance: an exploratory study. *Ital J Pediatr.* (2022) 23:48–60. 10.1186/s13052-022-01256-z 35461296 PMC9034068

[22] Street ME Sartori C Catellani C Righi B. Precocious puberty and COVID-19 into perspective: Potential increased frequency, possible causes, and a potential emergency to be addressed. *Front Pediatr.* (2021) 9:734899. 10.3389/fped.2021.734899 34616700 PMC8488256

[23] Kim JY Choe PG Oh Y Oh KJ Kim J Park SJ The first case of 2019 novel coronavirus pneumonia imported into Korea from Wuhan, China: implication for infection prevention and control measures. *J Korean Med Sci.* (2020) 35:e61. 10.3346/jkms.2020.35.e61 32030925 PMC7008073

[24] Roh SM Eun BW Seo JY. Does coronavirus disease 2019 affect body mass index of children and adolescents who visited a growth clinic in South Korea?: a single-center study. *Ann Pediatr Endocrinol Metab.* (2022) 27:52–9. 10.6065/apem.2142082.041 35038839 PMC8984750

[25] Park YJ Moon CM Yoo HJ. A study of factors influencing advanced puberty. *Korean J Pediatr* (2010) 53:146–51. 10.3345/kjp.2010.53.2.146

[26] Gwag SH Oh YR Ha JW Kang EG Nam HK Lee Y Weight changes of children in 1 year during COVID-19 pandemic. *J Pediatr Endocrinol Metab.* (2022) 35:297–302. 10.1515/jpem-2021-0554 34881539

[27] Korean Statistical Information Service. *Consumption of Health Functional Food.* (2021). Available online at: https://kosis.kr/statHtml/statHtml.do?orgId=145&tblId=DT_145010_009&conn_path=I2 (https://kosis.kr/statHtml/statHtml.do?orgId=145&tblId=DT_145010_009&conn_path=I2) (accessed March 10, 2022).

[28] Kim DH. Comparison of media usage behaviors of adolescents before and after COVID-19. *KISDI STAT Report.* (2021) 21:1–7.

[29] Yoon JY Yoon YH Yoon SL Lee WT. The current state of management and disposal of wastes related to COVID-19: A review. *J Korean Soc Environ Eng.* (2021) 43:739–46. 10.4491/ksee.2021.43.12.739

[30] Kaplowitz P Blosch C. Evaluation and referral of children with signs of early puberty. *Pediatrics.* (2016) 137:e20153732. 10.1542/peds.2015-3732 26668298

[31] Lee JS Kim JS Yang AR Cho SY Jin DK. Etiological trends in male central precocious puberty. *Ann Pediatr Endocrinol Metab.* (2018) 23:75–80. 10.6065/apem.2018.23.2.75 29969878 PMC6057022

[32] Park SC Trinh TA Lee WY Baek JY Lee SY Choi KH Effects of estrogen inhibition formula herbal mixture for danazol-induced precocious puberty in female rats: an experimental study with network pharmacology. *Integr Med Res.* (2021) 10:100708. 10.1016/j.imr.2020.100708 33665096 PMC7903350

